# STA-MCA bypass following sphenoid wing meningioma resection: A case report

**DOI:** 10.1016/j.ijscr.2019.05.025

**Published:** 2019-05-14

**Authors:** Anh Duc Nguyen, Tam Duc Le, Hung Manh Ngo, Hung Dinh Kieu

**Affiliations:** a Neurosurgery Department II, Viet Duc University Hospital, Hanoi, Viet Nam; b Neurosurgery Resident, Hanoi Medical University, Hanoi, Viet Nam; c Department of Neurosurgery and Spine Surgery, Hanoi Medical University Hospital, Hanoi, Viet Nam

**Keywords:** Sphenoid wing meningioma, MCA injury, STA-MCA bypass, Case report

## Abstract

**Introduction:**

Sphenoid meningioma engulfed cerebral arteries has always been a challenge. To achieve a gross total resection, vessel sacrifice may be unavoidable.

**Presentation of case:**

A 22-year-old man with a history of head trauma a week ago complained of a headache for one week. On examination, he was alert, denied paralysis and cranial nerves palsies. Preoperative MRI showed a hypervascular left sphenoid wing meningioma embedding left internal carotid artery and proximal segment of the middle cerebral artery. In operation, a branch of the MCA was divided when dissecting the tumor. The MCA was clipped but was still difficult to dissect vessel ends in the Sylvian fissure. We decided to extend craniotomy and did superficial temporal artery to M4 segment of MCA bypass. Then, the patient was resuscitated in surgical high dependency unit for 3 days. Surgical outcome in one year postoperative was good with KPS 90 out of 100 points and no neurological deficits. On postoperative MRA, STA-MCA bypass shown acceptable flow.

**Discussion:**

There were a few cases of skull base tumors requiring vessel revascularization. Most of the revascularization cases were meningiomas. Saphenous vein graft (SVGs) was the most commonly reported graft, followed by radial artery graft (RAGs). In case of difficulty in dissecting the vessel ends due to the tumor infiltration, STA-MCA bypass was a safe and helpful choice, especially the collateral vessels were present and the need for blood flow augmentation was minimal.

**Conclusion:**

STA-MCA bypass was effective surgical management for MCA injury in sphenoid wing meningioma resection.

## Highlights

• There were a few cases of skull base tumors requiring vessel revascularization. This is the first clinical report on this issue in Vietnam.
• Most of the vessel revascularization cases were meningiomas.
• Saphenous vein graft (SVGs) was the most commonly reported graft, followed by radial artery graft (RAGs).
• STA-MCA bypass was a safe and helpful choice, especially the collateral vessels were present and the need for blood flow augmentation was minimal.

## 1 Introduction

Complex skull base tumors have always been a challenge for complete resection because of their invasion to critical structures such as internal carotid arteries, main cerebral arteries, optic nerve, and cavernous sinus… Vessel injury may be unavoidable to perform the complete resection. Vessel revascularization should be considered if vessel sacrifice would cause symptomatic cerebral ischemia.

There were numerous cerebral revascularization techniques, which can be used for reconstruction after tumor resection. Choosing appropriate graft and bypass techniques depend on indications and variances in patient anatomy. Considered factors include the size of the recipient`s vessel, the desired amount of blood flow, the size and availability of donor`s vessels, the nature of the operation (emergent or elective), and the anatomy of the revascularization site and the pathology being treated [1,2].

There have not been yet any clinical reports on this issue in Vietnam before. This is because cerebral revascularization is a difficult technique for our country and the patients need to be evaluated carefully the blood supply of revascularization after removing the tumor. This article aims to report the first case emergent STA-MCA bypass due to MCA injury during sphenoid wing meningioma resection in Vietnam.

The work has been reported in line with the SCARE criteria [3].

## 2 Presentation of case

A 22-year-old man with a history of head trauma a week ago was admitted to our hospital with complaints of headache for one week. He had no nausea, vomiting and blurred vision. On examination, he was alert, oriented, but malaise. He denied of paralysis and cranial nerves palsies. His muscle strength was grade V.

**Fig. 1:**
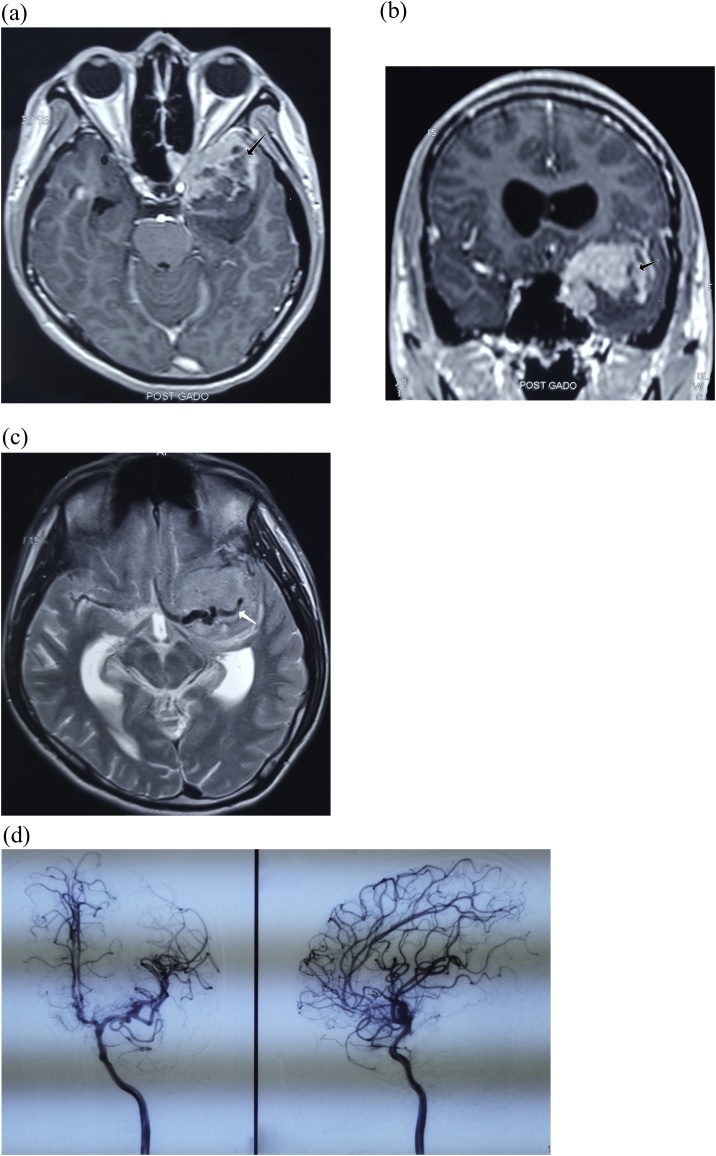
(A) and (B) Left sphenoid wing meningioma showed hypervascular and enhanced heterogeneously (black arrow). (C) The meningioma engulfed ICA and the proximal portion of MCA (white arrow). (D) On DSA, the branches of ICA and ECA on the same side fed the meningioma.

The preoperative MRI showed a hypervascular left sphenoid wing meningioma, which enhanced heterogeneously and embedded the intracranial portion of the left internal carotid artery and proximal segment of the middle cerebral artery (Fig. 1A, B and C). On DSA, the branches of ICA and ECA on the same side fed the meningioma (Fig. 1D and E).

The frontotemporal approach craniotomy was used. In operation, the tumor was hypervascular and infiltrated dura of inferior orbital fissure, temporal fossa and the MCA. A branch of the MCA was divided when dissecting the tumor. We clipped the MCA but it still was difficult to dissect its ends in the Sylvian fissure. We decided to extend craniotomy and did superficial temporal artery to M4 segment of MCA bypass. MCA was clipped for 45 min. Intraoperative blood loss was 1000 ml. The surgery took 7 h.

**Fig. 2:**
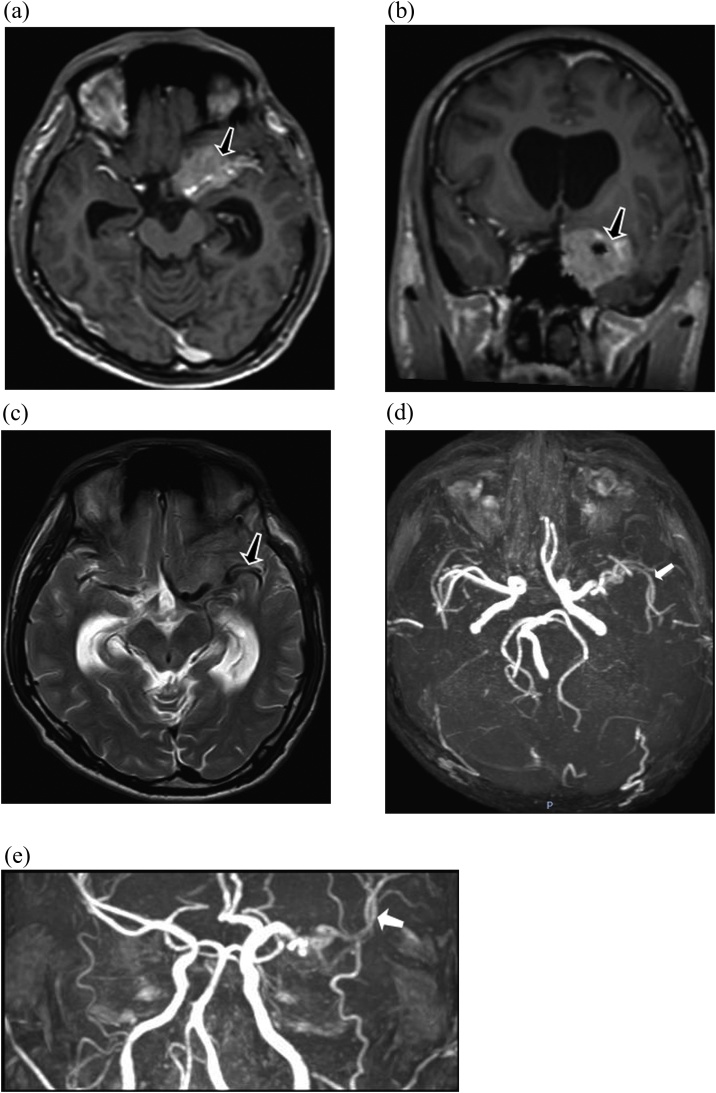
(A), (B) and (C). Post-operative left sphenoid meningioma enhanced and embedded ICA and MCA (black arrow). D and E, STA-MCA bypass had a good flow (white arrow).

After that, the patient was resuscitated in surgical high dependency unit for 3 days. Pathological findings proved transitional meningioma, WHO grade I. Surgical outcome in one year postoperative was good with KPS 90 out of 100 points. No neurosurgical deficits were reported. On MRA, STA-MCA bypass shown acceptable flow (Fig. 2D and E).

## 3 Discussion

There were a few cases of skull base tumors requiring vessel revascularization. In a recent systematic review, Wolfswinskel et al showed that only about 368 cases of EC-IC bypass due to vessel injury for skull base tumors resection were reported from 1950 to 2018 [4]. According to Sekhar, there were 130 revascularization cases for tumors from 1988 to 2006 [5]. The reason may be the increasing use of radiotherapy for tumor remnants left around vessels during surgery.

Skull base tumors include pituitary tumors, sellar/parasellar tumors, meningioma, chordomas, chondrosarcomas, and squamous cell carcinoma… Most of the vessel revascularization cases were meningiomas [4]. This is because of the variety in their behaviors resulting in different vessel encasement. Therefore, meningiomas resections often need revascularization. Most chordomas and chondrosarcomas can be dissected away from the vessel. However, complete resections of the slow-growing malignant tumors, such as adenoid cystic carcinomas, usually damage blood vessels.

Indications of cerebral revascularization in skull base tumors were still controversial. This depends on the nature of the tumors, history of radiotherapy, recurrent tumors and relation to nearby critical structures. Sekhar at al recommended four criteria for EC-IC bypass in the patients with skull base tumors [6]. In our case, this was an accidental intraoperative injury of the MCA and the artery cannot be repaired directly. Despite vessel sacrifice and revascularization, gross total resection was only achieved in 63% [5]. In Champagne`s study, complete resection occurred in 2 out of 12 cases (Simpson grades 2 and 3) [7].

In the previous studies, saphenous vein graft (SVGs) was the most commonly reported graft, followed by radial artery graft (RAGs) [5], [8], [9]. However, in our case, we cannot dissect the vessel ends due to the tumor infiltrated deeply vascular wall in the Sylvian fissure. Therefore, STA-MCA bypass was another good choice because of its safety and usefulness. Another reason why we decided to choose the STA-MCA bypass was that the collateral vessels were present and the need for blood flow augmentation was minimal.

## 4 Conclusion

Meningiomas, especially huge sphenoid wing ones, were the most common skull base tumors requiring revascularization. Despite the popularity of SVGs and RAGs, STA-MCA bypass was a safe and effective surgical management for vessel injury in sphenoid meningioma resection.

## Conflict of interest

Nothing to declare.

## Source of funding

This research did not receive any specific grant(s) from funding agencies in the public, commercial, or not-for-profit sectors.

## Ethical approval

Nothing to declare, as this is a single case report. At our center, we do not require ethical review by the Institutional Review Board for single case report studies.

## Consent

Written informed consent was obtained from the patient and his wife, for publication of this case report and accompanying images. A copy of the written consent is available for review by the Editor-in-Chief of this journal on request.

## Author contribution

• Anh Duc Nguyen: Helped with study concept, interpretation and writing the paper
• Tam Duc Le: Contributed to data collection and analysis, interpretation, writing the initial draft, editing and reviewing of the final manuscript and final submission of the paper.
• Hung Manh Ngo: Contributed to study concept and data collection.
• Hung Dinh Kieu: Contributed to conceptualization, supervision, and review of the final manuscript

## Registration of research studies

Not applicable – this is a single case report, not a systematic review or meta-analysis. Moreover, we attest that it is not a ‘first in man’ study, either.

## Guarantor

Anh Duc Nguyen.

## Provenance and peer review

Not commissioned, externally peer-reviewed.
